# Editorial: High Performance Cognition: Information-Processing in Complex Skills, Expert Performance, and Flow

**DOI:** 10.3389/fpsyg.2020.579950

**Published:** 2020-10-06

**Authors:** Benjamin Ultan Cowley, Frederic Dehais, Stephen Fairclough, Alexander John Karran, Jussi Palomäki, Otto Lappi

**Affiliations:** ^1^Faculty of Educational Sciences, University of Helsinki, Helsinki, Finland; ^2^Department of Digital Humanities, Faculty of Arts, University of Helsinki, Helsinki, Finland; ^3^Institut Supérieur de l'Aéronautique et de l'Espace (ISAE-SUPAERO), Toulouse, France; ^4^Liverpool John Moores University, Liverpool, United Kingdom; ^5^Hautes études Commerciales Montréal, Université de Montréal, Montreal, QC, Canada

**Keywords:** high performance cognition, cognitive neuroscience, expert performance, psychophysiology, flow, deliberate practice, cognitive fitness

In this Research Topic, High Performance Cognition (HPC) emerges as a generic term for the study of human performance and skill acquisition, from novices on an unfamiliar task (as most psychological experiments) to experts displaying superior levels of domain-specific skill. We received 11 pieces of empirical work across several experimental tasks and three articles describing theoretical/conceptual work on flow, cognitive training, and Deliberate Practice framework for expert performance. The papers received in this Research Topic encompassed a range of methodologies including reports of phenomenology, measurement of behavior, and psychological traits, and measures based on psychophysiology and neurophysiology.

What were the commonalities underlying the various contributions received for the topic? Most empirical papers report that the cognitive process under investigation showed interdepencies with other processes: from the longitudinal relationship between performance anticipation and Flow shown in Cowley et al., to the proactive control of executive function shown by “open”-skill athletes (i.e. trained in dynamic, externally-paced sports) in Yu et al.. This theme also reflects in theoretical contributions which call for revision of the longstanding constructs of deliberate practice and Flow, to either account for interdependency in a more holistic framework (Hambrick et al.), or control for it (Abuhamdeh), respectively. Clearly, even to make such revisions is challenging; beyond which is the yet more-challenging task to build a unified framework on the revised constructs, coherent with existing evidence. Yet such a framework will refine the generic term HPC to a definition of cognitive performance, and thereby provide grounds for empirical predictions and a direction for future work for many years.

Thus, while we cannot yet answer the question: “do cognitive processes that are specific to contexts of high performance also generalize across task domains?,” this Topic clarifies somewhat how we might start to address the question. A summary of the contributions follows to provide an overview of the specific articles received as part of this Research Topic.

## Empirical Studies

### Flow Focus

Chin and Kales investigate whether autonomic arousal (heart-rate variability, HRV) influences self-reported flow and performance in the Stroop task. To increase between-subjects HRV, participants first performed techniques in either nasal respiration, arm-muscle contraction, both combined, or read emotionally-neutral articles. The results showed an inverted-U pattern: performance and flow were maximal at moderate arousal levels. Optimal performance was also associated with predominantly sympathetic autonomic activity.

Cowley et al. ask: How does subjective feeling of high performance relate to development of (personally) optimal performance? They report a longitudinal (40 trials over 8 sessions) study, recording Flow and behavior as participants learned to perform a visuomotor steering task. Participants did not experience more Flow over sessions; instead, their trial-wise subjective anticipation of performance (estimated from a power-law model over trials) was strongly related to Flow.

Murch et al. report on three experiments which aim to identify objective markers of flow while gambling. The authors collected self-reported gambling flow and physiological data as measured by the cardiac pre-ejection period (PEP). The authors did not find evidence of changes in PEP when interacting with the electronic gambling machines but only interactions between subjective and objective measures during the first block of each experiment.

In a study examining the effect of positive feedback on perceived self-efficacy, flow and performance, Peifer et al. hypothesize that self-efficacy acts as a mediator, whereby positive feedback on one task imparts a positive effect upon performance and flow in subsequent tasks. They report evidence to support the hypothesis and propose multiple methods of intervention to further increase these positive effects.

Sinnett et al. ask: can increased flow experience can improve perception? The authors implemented a within-subjects design involving groups of athletes and musicians facing a temporal order judgement task paradigm. This task encompasses a measure of temporal processing measure and of spatial attention. Their results disclosed a positive relationship between the self-reported value of experienced flow and the efficiency of spatiotemporal information processing.

### Cognitive-Performance Focus

Here we order the studies by participant expertise, starting with naïve-subject designs.

Kee et al. investigate the effects of non-striving states, induced by a repetitive water-pouring task, on a word-length comparison task. The authors reported that the experimental group tended to exhibit lower performance and was significantly faster to perform the word comparison task than the control group. Though the authors did not use self-report questionnaires, they suggest their experimental design could help to improve mindfulness practice.

Prolonged task execution brings mental fatigue, characterized by “indolence, reduced motivation, and impaired performance.” Liu et al. investigate the motivating effect of monetary rewards (given during this low-vigilance state) on performance in a flanker task, and concomitant electrophysiological measures linked to selective visual attention, primarily P300. Monetary reward did improve performance during low vigilance, but neural measures recovered selectively, indicating that some constraints on higher cognitive performance are non-volitional.

Gong et al. investigate how EEG neurofeedback training (NFT) affects shooting performance and neuroplasticity among police students. They found that pre-post shooting performance increased for participants using sensorimotor-rhythm NFT, but slightly decreased for participants using alpha-rhythm NFT. Participants learned to alter their EEG patterns in both NFT groups. Pre-post measures also showed that neuroplasticity was affected by NFT. Thus, NFT may facilitate training for activities requiring sensorimotor accuracy.

Muñoz et al. present work toward a psychophysiological model of firearm training for real police officers using a combination of head-mounted virtual reality and non-intrusive physiological sensors. The authors demonstrate how changes in frontal theta activation and heart rate variability were affected by changes in task difficulty levels, which they articulate as metrics of “concentration” and “calmness.”

The study by Savostyanov et al. utilizes ERPs to examine the effect of long-term meditation on comprehension of statements about oneself and others. Three groups (control, short- and long-term meditators) completed a reading task containing self or non-self-related evocative statements and grammatical errors. They demonstrate that meditation increases negative affect processing and an ERP analysis that potentially shows an increase of voluntary control over emotional states for meditators.

Work presented by Yu et al. investigates how motor-skills experience modulates proactive and reactive control of executive function, using a cued task-switching protocol with “open”- and “closed”-skilled athletes and non-athlete controls. Their findings highlight that open-skilled participants showed significantly less positive-going parietal cue-locked P3 and better predictive task-switching performance. They conclude that proactive control may be enhanced in open-skilled participants when compared to the other groups.

## Theoretical Papers

Aidman proposes a framework for Cognitive Fitness: trainable cognitive abilities (as distinct from domain-specific knowledge, skills and attitudes) that can be improved and maintained by specific cognitive fitness training, analogous to physical-fitness training for strength, endurance, and flexibility. Aidman proposes his framework would be valuable for training performance in sport, arts, emergency services—indeed, any domain where individuals must deliver high performance on demand under stressful conditions.

Hambrick et al. and Abuhamdeh present critical opinion pieces on two highly-influential theoretical constructs in study of HPC: Deliberate Practice (DP, Ericsson et al., 1993), and Flow (Csikszentmihalyi, 1975). The Hambrick et al. paper continues their ongoing interrogation of the internal consistency of existing DP research, building on the authors' earlier meta-analysis (Macnamara et al., 2014). The Abuhamdeh paper looks critically at how the original definition of flow has since been operationally defined.

Both contributions share an underlying message: after decades of research, both lines of investigation remain pre-paradigmatic, in the sense of Kuhn (1962). Each is identified with one researcher's seminal account, but subsequently has been operationalized in an unsystematic way. This inconsistency, alongside lack of agreement on theoretical attributes, threatens the ability of both theoretical perspectives to develop into mature paradigms. Both papers challenge researchers to unify methodology and theory, and develop “progressive research programs” (Lakatos, 1978).

## Summary

The studies presented in this Research Topic illustrate both current diversity, and a bright future, for research in performance cognition. Researchers investigating flow states posit new experiments to model the conditions and relationships involved and call for a consistent operationalization of the flow state. Neurofeedback researchers propose to move into real-time adaptation of training protocols; those investigating meditation propose novel interventions to further increase the benefits of the practice. Extension and improvement of human cognition through augmented and directed training comprises a common vision for the future. To achieve it, we must unify this diverse field around a common and parsimonious framework, such that we can move toward measuring and understanding high performance cognition as it develops through learning and training across various domains.

## Author Contributions

All authors listed have made a substantial, direct and intellectual contribution to the work, and approved it for publication.

## Conflict of Interest

The authors declare that the research was conducted in the absence of any commercial or financial relationships that could be construed as a potential conflict of interest.
